# Evaluation of 3 Electronic Methods Used to Detect Influenza Diagnoses during 2009 Pandemic

**DOI:** 10.3201/eid1912.131012

**Published:** 2013-12

**Authors:** Sunita Mulpuru, Tiffany Smith, Nadine Lawrence, Kumanan Wilson, Alan J Forster

**Affiliations:** University of Ottawa, Ottawa, Ontario, Canada (S. Mulpuru, K. Wilson, A.J. Forster);; Public Health Agency of Canada, Ottawa (T. Smith);; Ottawa Hospital Research Institute, Ottawa (N. Lawrence, K. Wilson, A.J. Forster)

**Keywords:** influenza, ICD-10, database, diagnosis, pandemic, influenza A(H1N1)pdm09, viruses, Canada

**To the Editor:** Conducting influenza surveillance in hospitals is imperative to detect outbreaks, inform infection control policy, and allocate resources (*1*). Hospital administrative data could be harnessed for this purpose (*2*,*3*) but are not currently used for infection surveillance because of data lag times. Influenza cases could be identified by using International Classification of Diseases, Tenth Revision, Clinical Modification (ICD-10-CM), codes within the discharge abstract, pharmacy, and microbiology laboratory information systems. Although these approaches are assumed to accurately identify influenza cases, this assumption has not been widely tested, especially during a pandemic. In this retrospective cohort study, we aimed to identify and evaluate 3 electronic methods of influenza case detection during 1 peak of influenza A(H1N1)pdm09.

With ethics board approval, we used the Ottawa Hospital Data Warehouse (OHDW) (Ottawa, ON, Canada) to identify 398 adult inpatients at the Ottawa Hospital during October–December 2009 who had cardiac, infectious, or respiratory disease diagnoses (ICD-10-CM codes: all J codes, A15–19, A37, A40, A41, A49, I26, I28, I50, I51.4, R57). OHDW is a relational database containing pharmacy, laboratory, and discharge diagnosis information for inpatients at Ottawa Hospital. We detected influenza in the following ways: influenza diagnosis in the discharge abstract database (DAD) (ICD-10-CM codes J09–J11); prescription for an antiviral drug (oseltamivir, zanamivir) in the pharmacy system; and a positive laboratory test during the hospital encounter (without specifying test type or specimen) in the laboratory system.

We assessed these case definitions against a criterion standard of influenza diagnosis on the hospital chart, determined by a physician reviewer blinded to the electronic values for the case definitions. We constructed 2 × 2 contingency tables for each classification method and calculated sensitivity, specificity, positive predictive value (PPV), and likelihood ratios using standard equations.

Influenza prevalence in this cohort was 13.6% (54/398) by our criterion standard. The proportion of male and female patients was equal, with a median age of 69 years (interquartile range 53–81 years). Median length of hospital stay was 6 days (interquartile range 1–12 days). A total of 77 (19.3%) patients were admitted to the intensive care unit, and 51 (12.8%) patients died in hospital. Two (0.5%) patients died with a primary diagnosis of influenza. The Table shows the performance characteristics of each influenza classification method against the criterion standard. The DAD-based influenza diagnosis algorithm was most accurate, with sensitivity of 90.7% (95% CI 79.7%–96.9%), specificity of 96.5% (95% CI 94%–98.2%), and PPV of 80.3%.

**Table:** Performance characteristics of electronic influenza classification methods compared to criterion standard chart review, Ottawa Hospital, Ottawa, Ontario, Canada, October–December 2009*

Influenza classification method	No.					% (95% CI)			%			% (95% CI)	
	TP	FP	TN	FN		Sensitivity	Specificity		PPV	NPV		PLR	NLR
DAD flu diagnosis	49	12	332	5		90.7 (79.7–96.9)	96.5 (94–98.2)		80.3	98.5		26 (14.9–45.7)	0.10 (0.04–0.22)
Positive laboratory result	43	7	337	11		79.7 (66.5–89.4)	98 (95.9–99.2)		86.0	96.8		39.1 (18.6–82.5)	0.21 (0.12–0.35)
Antiviral drug prescribed	51	83	261	3		94.4 (84.6–98.8)	75.9 (71–80.3)		38.0	98.8		3.9 (3.2–4.8)	0.07 (0.02–0.22)

*TP, true positive; FP, false positive; TN, true negative: FN, false negative; PPV, positive predictive value; NPV, negative predictive value; PLR, positive likelihood ratio; NLR, negative likelihood ratio; DAD Flu Diagnosis = International Classification of Diseases, Tenth Revision, Clinical Modification, diagnosis code for influenza on the discharge abstract database stored in the Ottawa Hospital Data Warehouse.

Our results demonstrate adequate correlation between ICD-10-CM coding for influenza in adults during a 3-month peak of the pandemic season within a single institution. Coding or interpretative errors were the probable cause of the 10% false-negative and 3% false-positive rates of ICD-10 coding for influenza on the DAD.

Classifying influenza by antiviral prescription was sensitive but less specific than clinical diagnosis. This finding could be explained by empiric antiviral prescriptions for infectious respiratory symptoms being written before confirmatory testing (*4*). Influenza classification by positive laboratory tests was specific but less sensitive in this analysis, probably because of nonuniform laboratory testing among inpatients from lack of specific criteria to guide testing and lack of testing in those with less severe illness. Not all patients who have influenza are tested for it, and these diagnoses would be classed as false negatives, influencing the sensitivity downward. Furthermore, laboratory testing would be likely to miss patients with influenza-triggered exacerbations of congestive heart failure and chronic obstructive pulmonary disease (*5*), which would underestimate influenza cases.

Our study correlated ICD-10-CM–specific codes for influenza in hospitalized adults during 1 peak of the 2009 influenza pandemic. A previous study in the United States in 2006 evaluated ICD-9-CM admission and discharge influenza codes in hospitalized children (*6*). The authors found that of 715 laboratory-confirmed influenza cases, ICD-9-CM codes were only 65% sensitive, suggesting that use of these codes for surveillance would underestimate influenza hospitalizations by 35% (*6*). This work was undertaken in 3 consecutive nonpandemic influenza seasons during 2001–2004.

Our findings must be generalized with caution because our study evaluated ICD-10-CM coding accuracy over 3 months of a pandemic influenza season in adults at 1 academic hospital. With lower influenza prevalence, the PPV would drop, suggesting that the coded diagnosis would overestimate influenza hospitalizations. Furthermore, sensitivity and specificity of codes might not be static measures because the diagnosis of influenza on the chart might be influenced by the prevalence of influenza in communities (*7*).

Given these limitations, further work is needed to fully validate ICD-10 codes for influenza during seasons of low prevalence and in other populations including children. Despite this, our results have implications for future research using administrative data to develop timely surveillance systems, track costs, and monitor resource use.
